# Supplementary material for: The Antiviral Effects of Heat-Killed Lactococcus lactis Strain Plasma Against Dengue, Chikungunya, and Zika Viruses in Humans by Upregulating the IFN-α Signaling Pathway
Source: Microorganisms. 2024 Nov 13;12(11):2304. doi: 10.3390/microorganisms12112304 (PMC11596828; doi:10.3390/microorganisms12112304)
Supplement: Supplementary file 1 [file microorganisms-12-02304-s001.zip › microorganisms-3287471-supplementary.pdf]

Supplementary materials

Table S1. The antiviral effect of recombinant IFN-α (100U), LCP Sup 1:10, and CpG Sup 1:10, were evaluated based on the log reduction normalized to Neg Sup 1:10 (median ± SD) N=5. FFURA= Foci forming unit reduction assay, PFURA= Plaque forming unit reduction assay, IFN-α (100 U) = interferon alpha 100 units, CpG Sup 1:10= 10-fold diluted supernatants of CpG ODN 2216-stimulated PBMC, LCP Sup 1:10= 10-fold diluted supernatants of LC-Plasma-stimulated PBMC, LCP Sup 1:100= 100-fold diluted supernatants of LC-Plasma-stimulated PBMC, and LCP Sup 1:1000= 1000-fold diluted supernatants of LC-Plasma-stimulated PBMCs.

	Anti-DENV		Anti-CHIKV		Anti-ZIKV	
Treatment	No. of log reduction (FFURA)	No. of log reduction (qRT-PCR)	No. of log reduction (PFURA)	No. of log reduction (qRT-PCR)	No. of log reduction (PFURA)	No. of log reduction (qRT-PCR)
IFN-α (100 U)	4.86 ± 0.77	2.95 ± 0.55	6.11 ± 0.97	4.49 ± 0.53	5.15 ± 1.27	3.34 ± 0.44
CpG Sup 1:10	5.63 ± 2.02	3.37 ± 0.88	6.48 ± 1.53	4.97 ± 0.32	3.95 ± 2.00	4.23 ± 0.74
LCP Sup 1:10	2.15 ± 0.67	1.50 ± 0.71	3.44 ± 2.36	2.76 ± 1.60	2.51 ± 0.64	1.53 ± 0.77
LCP Sup 1:100	0.99 ± 0.70	0.59 ± 0.22	1.15 ± 0.39	0.44 ± 0.33	2.00 ± 01.11	0.69 ± 0.47
LCP Sup 1:1000	0.15 ± 0.43	0.18 ± 0.28	0.97 ± 0.59	0.34 ± 0.32	1.09 ± 1.22	0.40 ± 0.47

Table S2. Fold-increase in ISGs of Huh-7 cells following treatments with recombinant IFN-α (100 units), CpG Sup 1:10, and LCP Sup 1:10, (median ± SD) N=5.

ISGs	Fold-increase in Interferon stimulative genes			
	IFN-α (100 U)	CpG Sup 1:10	LCP Sup 1:10	Neg Sup 1:10
IFITM-1	20.56 ± 8.86	15.43 ± 6.42	9.73 ± 5.87	1.31 ± 0.37
ISG15	77.3 ± 37.5	63.90 ± 51.00	10.5 ± 4.13	1.62 ± 0.62
ISG20	7.12 ± 5.82	5.54 ± 5.11	3.52 ± 1.18	1.21 ± 0.35
Mx-A	254.70 ± 45.80	278.0 ± 60.83	23.11 ± 63.14	1.160 ± 2.140
OAS-1	75.11 ± 42.44	60.25 ± 41.50	12.01 ± 16.14	1.69 ± 3.16
RSAD2	4486 ± 6115	2678 ± 5666	17.1 ± 69.1	0.58 ± 0.31
RYDEN	21.42 ± 24.74	23.92 ± 36.16	8.04 ± 4.80	1.930 ± 7.60
